# Characteristics and risk factors for 28-day mortality of hospital acquired fungemias in ICUs: data from the EUROBACT study

**DOI:** 10.1186/s13054-016-1229-1

**Published:** 2016-03-09

**Authors:** José-Artur Paiva, José Manuel Pereira, Alexis Tabah, Adam Mikstacki, Frederico Bruzzi de Carvalho, Despoina Koulenti, Stéphane Ruckly, Nahit Çakar, Benoit Misset, George Dimopoulos, Massimo Antonelli, Jordi Rello, Xiaochun Ma, Barbara Tamowicz, Jean-François Timsit

**Affiliations:** Grupo de Infecção e Sepsis; Emergency and Intensive Care Department, Centro Hospitalar S. João, EPE; Faculty of Medicine, University of Porto, Porto, Portugal; Université Grenoble 1, U 823, Albert Bonniot Institute; Team 11: Outcome of mechanically ventilated patients and respiratory cancers, Grenoble, France; Burns, Trauma, and Critical Care Research Center, The University of Queensland, Butterfield Street, Brisbane, Australia; Outcomerea Organization, Paris, France; Department of Anaesthesiology and Intensive Therapy, Regional Hospital in Poznan, Poznan University of Medical Sciences, Poznan, Poland; Infectious and Tropical Diseases Intensive Care Unit, Hospital Eduardo de Menezes, Fundação Hospitalar do Estado de Minas Gerais, Belo Horizonte, MG Brazil; Department of Critical Care, University Hospital ATTIKON, Medical School University of Athens, Athens, Greece; Decision Sciences in Infectious Disease (DescID) Prevention, Control and Care, UMR 1137 Paris Diderot University, Sorbonne, Paris, France; Department of Anaesthesiology and Intensive Care, Istanbul University and Istanbul Medical School, Istanbul, Turkey; Université Paris Descartes, Paris Sorbonne Cité, Medical-surgical ICU, Groupe hospitalier Paris Saint-Joseph, Paris, France; Department of Intensive Care and Anaesthesiology, Policlinico Universitario A. Gemelli-Università Cattolica del Sacro Cuore, Rome, Italy; Critical Care Department, Hospital Vall d’Hebron, Universitat Autònoma de Barcelona, Barcelona, Spain; Department of Critical Care Medicine, The First Affiliated Hospital Of China Medical University, Shenyang, China; Medical and Infectious Diseases Intensive Care Unit, Bichat University Hospital, Paris Diderot University, Paris, 75018 France

## Abstract

**Background:**

To characterize and identify prognostic factors for 28-day mortality among patients with hospital-acquired fungemia (HAF) in the Intensive Care Unit (ICU).

**Methods:**

A sub-analysis of a prospective, multicenter non-representative cohort study conducted in 162 ICUs in 24 countries.

**Results:**

Of the 1156 patients with hospital-acquired bloodstream infections (HA-BSI) included in the EUROBACT study, 96 patients had a HAF. Median time to its diagnosis was 20 days (IQR 10.5–30.5) and 9 days (IQR 3–15.5) after hospital and ICU admission, respectively. Median time to positivity of blood culture was longer in fungemia than in bacteremia (48.7 h vs. 38.1 h; *p* = 0.0004). *Candida albicans* was the most frequent fungus isolated (57.1 %), followed by *Candida glabrata* (15.3 %) and *Candida parapsilosis* (10.2 %). No clear source of HAF was detected in 33.3 % of the episodes and it was catheter-related in 21.9 % of them. Compared to patients with bacteremia, HAF patients had a higher rate of septic shock (39.6 % vs. 21.6 %; *p* = 0.0003) and renal dysfunction (25 % vs. 12.4 %; *p* = 0.0023) on admission and a higher rate of renal failure (26 % vs. 16.2 %; *p* = 0.0273) at diagnosis. Adequate treatment started within 24 h after blood culture collection was less frequent in HAF patients (22.9 % vs. 55.3 %; *p* < 0.001). The 28-day all cause fatality was 40.6 %. According to multivariate analysis, only liver failure (OR 14.35; 95 % CI 1.17–175.6; *p* = 0.037), need for mechanical ventilation (OR 8.86; 95 % CI 1.2–65.24; *p* = 0.032) and ICU admission for medical reason (OR 3.87; 95 % CI 1.25–11.99; *p* = 0.020) were independent predictors of 28-day mortality in HAF patients.

**Conclusions:**

Fungi are an important cause of hospital-acquired BSI in the ICU. Patients with HAF present more frequently with septic shock and renal dysfunction on ICU admission and have a higher rate of renal failure at diagnosis. HAF are associated with a significant 28-day mortality rate (40 %), but delayed adequate antifungal therapy was not an independent risk factor for death. Liver failure, need for mechanical ventilation and ICU admission for medical reason were the only independent predictors of 28-day mortality.

**Electronic supplementary material:**

The online version of this article (doi:10.1186/s13054-016-1229-1) contains supplementary material, which is available to authorized users.

## Background

Bloodstream infection(s) (BSI) in the critically ill patients are a major cause of morbidity and mortality. The prevalence varies between centers, representing 15 % of all nosocomial infections in a recent, large, multicenter prevalence study [1]. The prognosis of BSI also varies, depending on several factors related to the host, the pathogen and the antimicrobial agent.

Fungi are responsible for around 20 % of all microbiologically documented infections in the Intensive Care Unit (ICU) [1]. The incidence of invasive fungal infections has increased steadily, namely due to the increasing number of both immunocompromised and critically ill patients. In the last decades, we faced a worldwide rise in the prevalence of candidemia, particularly in the ICU [2–6]. Data from surveillance programs from the USA and Europe showed that *Candida* spp. is responsible for 2–11 % of hospital-acquired BSI (HA-BSI) and it represents 8.3 % of patients with HA-BSI hospitalized in ICUs [7]. It is the fourth cause of nosocomial BSI in the USA and the fifth to the tenth most common pathogen in Europe [8–11].

Candidemia is a severe disease linked to significant morbidity and mortality [3, 12, 13] ranging from 35–75 % [14, 15]. Outstandingly, after controlling for confounders, candidemia has been identified as an independent predictor of mortality [16]. In addition, it prolongs hospital length of stay and increases costs associated with patient management [13, 17]. Therefore, it is important to identify potentially modifiable prognostic factors to improve this poor outcome. Few independent prognostic factors have been identified in critically ill patients with candidemia. Adequate initial therapy is of paramount importance for a successful outcome. In general, early administration of antimicrobial agents is associated with a better outcome [18]. However, contradictory results have been published on the timing of antifungal therapy [19].

The goal of this sub-analysis of the Epidemiology and outcome of hospital-acquired bacteremia (EUROBACT) study was to characterize the population of patients with hospital-acquired fungemia (HAF) admitted to ICUs worldwide and to identify prognostic factors for 28-day mortality, including timing of antifungal therapy, in these patients.

## Methods

A prospective observational multicenter international cohort design was used. The international database was declared to the CNIL (Commission Nationale de l’Informatique et des Libertés). The French ethics committee waived the need for informed consent for French centers. Similar authorization was obtained from countries such as Portugal (Centro Hospitalar S. João), Poland (Poznan University of Medical Sciences) and Australia (Royal Brisbane and Women’s Hospital) and it was waived in the other countries due to the observational nature of the study.

### Study protocol and definitions

Patients were enrolled if they had a new diagnosis of HA-BSI and were admitted to an ICU. The study focused on the first episode of HA-BSI, either being ICU-acquired or acquired before admission to ICU. The detailed protocol has been described previously [7]. Data collected for each patient included the dates and times of collection and of positivity of the first positive blood culture; source of infection; presence of sepsis; severity of illness; comorbidities; and infection management, including source control, antimicrobial drugs and adjunctive treatments. Organ dysfunction and organ failure were defined as Sequential Organ Failure Assessment (SOFA) scores >0 and ≥3, respectively. All study data were obtained from patient files, and no additional tests were performed for the purpose of the study. Severity of illness was defined at ICU admission using the Simplified Acute Physiology Score (SAPS) II [20] and at HA-BSI diagnosis using the SOFA score [21]. Comorbidities were assessed using the Charlson index and the five markers of the Chronic Health Evaluation from the Acute Physiology and Chronic Health Evaluation (APACHE) II score, as reported by Knaus et al. [22]. Clinical variables and relapses or new episodes of HA-BSI were recorded until ICU discharge, and the all-cause mortality within 28 days of the first positive blood culture were ascertained.

### Data management and statistical analysis

A control quality check has been detailed previously [7]. The statistical analysis was based only on the first episode of HA-BSI, as this was the only episode for which full information was available. The medians and interquartile range (IQR) was computed for continuous data and Fisher’s exact test or the chi-square test was performed to compare categorical data. We compared characteristics of patients with bacteremia and patients with fungemia, using univariate hierarchical logistic regression models, including random effects for country and center. Time to death was plotted using Kaplan–Meier curves and compared using a frailty Cox model, treating the center as a random effect.

For patients with fungemia, risk factors for death were analyzed using hierarchical models. The variables were organized into three tiers: country, ICU and patient. To identify factors associated with day-28 mortality, we built a three-tiered hierarchical logistic mixed model using the GLIMMIX procedure in the SAS software. The influence of country-based and ICU-based variables on the outcome was included through both fixed and random effects. Multilevel modeling takes into account the hierarchical structure of the data, which may manifest as intra-class correlations. To obtain a conservative estimate of the standard error, a separate random-error term should be specified for each level of the analysis. Therefore, to avoid overestimating the significance of risk factors for death by day 28, we took intra-class correlation into account, and we specified a separate random-error term for each tier. Variables potentially associated with day-28 mortality (*p* values <0.20 by univariate analysis) were introduced into the multivariable model and selected using a backward approach. Two-way clinically relevant interactions were tested in the final model. In all analysis, two-sided *p* values <0.05 were deemed statistically significant. No correction for multiple testing was performed.

## Results

A fungus was recovered from the blood in 96 of the 1,156 patients (8.3 %) with HA-BSI admitted to an ICU, included in the EUROBACT study [7]. Patients with HAF were mostly male (67.7 %) with a median age of 61 years (IQR 48–73) and a median SAPS II score of 49 (IQR 41–63) (Table 1). They were mostly admitted to the ICU for medical reasons (63.5 %), 60 % of these with acute respiratory failure requiring ventilator support and 24 % with a cardiac-related syndrome: 35 % of the patients had a Charlson co-morbidity index ≥3. A chronic illness was present in 41.7 % of patients, mainly immunosuppressive (15.6 %), cardiovascular (14.6 %) and respiratory (12.5 %) chronic illnesses.

**Table 1:** Baseline characteristics of patients with fungemia and patients with bacteremia

Variable	Bacteremia patients (n = 1,060)	Fungemia patients (n = 96)	*P* value
Age, median (IQR)	62 (49–74)	61 (48–73.5)	0.7077
Male gender, n (%)	691 (65.2)	65 (67.7)	0.6155
Simplified Acute Physiology Score II, median (IQR)	48 (38–59)	49 (41–63)	0.0993
Medical admission, n (%)	611 (57.6)	61 (63.5)	0.1799
Charlson comorbidity index score, n (%)			0.3215
0	361 (34.1)	25 (26)	
1–2	367 (34.6)	37 (38.5)	
≥3	332 (31.3)	34 (35.4)	
At least one chronic illness, n (%)	336 (31.7)	40 (41.7)	0.0629
Immunosuppression, n (%)	136 (12.8)	15 (15.6)	0.5012
Cardiovascular, n (%)	103 (9.7)	14 (14.6)	0.202
Respiratory, n (%)	86 (8.1)	12 (12.5)	0.1286
Renal, n (%)	53 (5)	8 (8.3)	0.1396
Liver, n (%)	40 (3.8)	4 (4.2)	0.9752
At least one organ dysfunction on admission, n (%)	944 (89.1)	86 (89.6)	0.8491
Cardiovascular, n (%)	530 (50)	61 (63.5)	0.0222
Respiratory, n (%)	858 (80.9)	79 (82.3)	0.7319
Neurologic, n (%)	326 (30.8)	28 (29.2)	0.8062
Renal, n (%)	131 (12.4)	24 (25)	0.0023
Septic shock at admission, n (%)	229 (21.6)	38 (39.6)	0.0003
Mortality at 28 days, n (%)	375 (35.4)	39 (40.6)	0.2307

At ICU admission, at least one organ failure was documented in a significant number of patients (89.6 %). Respiratory (82.3 %) and cardiovascular (63.5 %) were the most prevalent organ dysfunctions followed by neurological (29.2 %) and renal (25 %). Septic shock was diagnosed in 39.6 % of the patients and it was present at diagnosis of HAF in half of the episodes.

The median time to diagnosis of HAF was 20 days (IQR 10.5–30.5) after hospital admission and 9 days (IQR 3–15.5) after ICU admission (Table 2). In fact, more than half of the episodes (56.3 %) occurred after the first week in the ICU. However, a significant number of patients (22.9 %) had already presented with HAF on ICU admission.

**Table 2:** Characteristics of fungemia and bacteremia episodes at diagnosis

Variable	Bacteremia patients (n = 1,060)	Fungemia patients (n = 96)	*P* value
Delay between ICU admission and BSI, days, median (IQR)	8 (3–16)	9 (3–15.5)	0.9812
Delay between hospital admission and BSI, days, median (IQR)	13 (7–25)	20 (10.5–30.5)	0.1299
Delay to positivity of the blood culture sampling, days, median (IQR)	38.1 (21.1–69.2)	48.7 (33–81)	0.0004
Sepsis syndrome			0.5420
Sepsis, n (%)	136 (12.8)	14 (14.6)	
Severe sepsis, n (%)	442 (41.7)	34 (35.4)	
Septic shock, n (%)	482 (45.5)	48 (50)	
SOFA score			0.4812
0–4, n (%)	256 (24.2)	17 (17.7)	
5–7, n (%)	284 (26.8)	24 (25)	
8–11, n (%)	300 (28.3)	31 (32.3)	
≥12, n (%)	220 (20.8)	24 (25)	
SOFA respiratory ≥3, n (%)	499 (47.1)	45 (46.9)	0.9385
SOFA cardiovascular ≥3, n (%)	441 (41.6)	47 (49)	0.2493
SOFA neurological ≥3, n (%)	340 (32.1)	32 (33.3)	0.6578
SOFA renal ≥3, n (%)	172 (16.2)	25 (26)	0.0273
SOFA coagulation ≥3, n (%)	134 (12.6)	15 (15.6)	0.5577
SOFA liver ≥3, n (%)	81 (7.6)	7 (7.3)	0.7845
Need for mechanical ventilation, n (%)	946 (89.2)	84 (87.5)	0.6661
Hypotension, n (%)	529 (49.9)	54 (56.3)	0.3263
Presumed source of infection			0.3336
No clear source, n (%)	242 (22.8)	32 (33.3)	
Catheter-related, n (%)	226 (21.3)	21 (21.9)	
Respiratory, n (%)	230 (21.7)	14 (14.6)	
Intra-abdominal, n (%)	122 (11.5)	12 (12.5)	
Urinary, n (%)	43 (4.1)	4 (4.2)	
Others, n (%)	67 (6.3)	6 (6.3)	
Multiple sources, n (%)	130 (12.3)	7 (7.3)	
Source control			0.9820
Not required, n (%)	590 (55.7)	54 (56.3)	
Done, n (%)	429 (40.5)	38 (39.6)	
Required not done, n (%)	41 (3.9)	4 (4.2)	
Delay of adequate treatment			<0.0001
<24 h	586 (55.3)	22 (22.9)	
>24 h and ≤120 h	335 (31.6)	59 (61.5)	
>120 h	40 (3.8)	9 (9.4)	
Never	99 (9.3)	6 (6.2)	

*SOFA* Sequential Organ Failure Assessment

Median time to blood culture positivity in fungemia was 48.7 hours (IQR 33–81). There was no clear source of fungemia in 32 patients (33.3 %) and in 21 patients (21.9 %) it was catheter-related, based upon culture results yielding identical microorganisms. The abdomen was the source of infection in 12 patients (12.5 %) (Table 2). Source control was required in 43.8 % of patients and was performed in 39.6 % of the patients (Table 2).

**Fig. 1:**
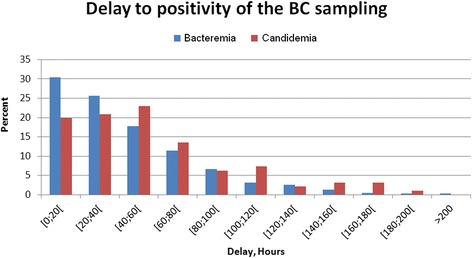
Delay to positivity of culture sampling. BC - Blood cultures

**Fig. 2:**
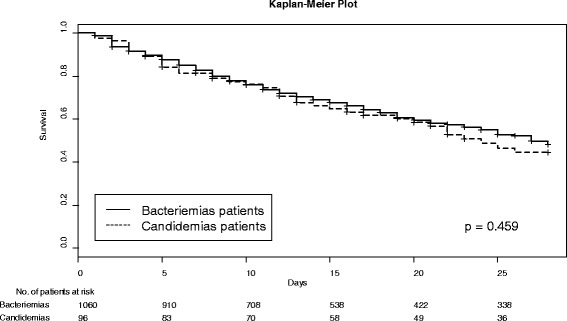
Time to death for fungemic and bacteremic patients

The characteristics on admission of patients with fungemia and of those with bacteremia were similar except for a higher rate of septic shock and renal dysfunction in fungemic patients (Table 1). At diagnosis, patients with HAF presented with a significantly higher rate of renal failure than bacteremic patients. Delay from hospital admission to HA-BSI diagnosis was not significantly different between bacteremic and fungemic patients (13 days (7–25) vs 20 days (10.5–30.5), *p* = 0.13). Delay of positivity of culture sampling was significantly longer for fungemia (38.1 hours (21.1–69.2) vs 48.7 (33–81), *p* = 0.0004) (Table 2 and Fig. 1). There was no significant difference between these two groups of patients in time to death (Fig. 2).

On comparing baseline characteristics in patients with ICU-acquired fungemia (n = 74) and HAF (n = 22), the only difference was more frequent presence of at least one chronic illness (*p* <0.01), namely immunosupression (*p* <0.01) and cardiovascular disease (*p* = 0,05) in patients with HAF (Additional file 1: Table S1). The only significant difference in the characteristics of fungemia episodes at diagnosis, was a higher coagulation SOFA score in patients with HAF (*p* <0.01) (Additional file 1: Table S2).

*Candida albicans* was the most frequent fungus isolated (57.1 %) followed by *Candida glabrata* (15.3 %) and *Candida parapsilosis* (10.2 %) (Table 3). Non-albicans *Candida* accounted for 39.6 % of HAF in this study. More than one fungus was recovered from the blood of two of the patients. A mixed (bacterial and fungal) infection was documented in 15.6 % of the patients with fungemia.

**Table 3:** Distribution of the 98 fungal pathogens

Pathogen	All fungemia	ICU acquired fungemia patients	Hospital acquired fungemia patients
	n = 98 (100 %)	n = 75 (76.5 %)	n = 23 (23.5 %)
*Candida albicans*, n (%)	56 (57,1)	44 (58.7)	12 (52.7)
*Candida glabrata*, n (%)	15 (15,3)	12 (16.0)	3 (13.0)
*Candida parapsilosis*, n (%)	10 (10,2)	7 (9.3)	3 (13.0)
Other Candida, n (%)	7 (7.1)	5 (6.7)	2 (8.7)
*Candida tropicalis*, n (%)	6 (6.1)	4 (5.3)	2 (8.7)
Other fungi, n (%)	3 (3.1)	2 (2.7)	1 (4.4)
*Candida krusei*, n (%)	1 (1.0)	1 (1.3)	0

In this study, 93.8 % of the patients received adequate treatment: 22.9 % of the patients received adequate treatment in the first 24 hours after blood culture collection, which was a significantly lower percentage than for bacteremia (55.3 %, *p* <0.001).

The 28-day all-cause mortality was 40.6 % (n = 39), slightly higher than in bacteremia (35.4 %). Non-survivors were older (64 years, IQR 50–76 vs 57 years IQR 46–71, *p* = 0.318) and had higher SAPS II score (52 IQR 43–63 vs 47 IQR 37–63, *p* = 0.095) but the differences were not statistically significant (Table 4). Fungemic patients admitted for a medical reason had significantly higher 28-day mortality (76.9 % vs 54.4 %, *p* = 0.038). Neither the Charlson comorbidity index nor the presence of a chronic illness had a significant impact on the outcome. No association was found between sepsis syndrome severity at HAF diagnosis and mortality. In univariate analysis, the SOFA score at HA-BSI diagnosis was associated with a worse outcome. A SOFA score ≥8 was more frequent in non-survivors (77 % vs 43.8 %, *p* = 0.004). Furthermore, non-survivors had significantly higher SOFA scores for cardiovascular organ (*p* = 0.015) and respiratory organ (*p* = 0.0019) dysfunction. The presumed source of infection and the pathogen did not affect 28-day mortality. Delay of adequate treatment also did not affect outcome (Table 4). The proportion of patients who received adequate antifungal therapy in the first 24 hours after positive blood culture collection was higher (though not statistically significant) in non-survivors than that in survivors (28.2 % vs. 19.3 %; *p* = 0.694).

**Table 4:** Risk factors for death at 28 days in the subpopulation with candidemia (univariate analysis)

Variable	Dead (n = 39 patients)	Alive (n = 57 patients)	*P* value
Age, median (IQR)	64 (50–76)	57 (46–71)	0.318
SAPS II, median (IQR)	52 (43–63)	47 (37–63)	0.095
Male, n (%)	28 (71.8)	37 (64.9)	0.448
Medical admission, n (%)	30 (76.9)	31 (54.4)	0.038
Charlson comorbidity index score, n (%)			0.5816
0	8 (20.5)	17 (29.8)	
1–2	17 (43.6)	20 (35.1)	
≥3	14 (35.9)	20 (35.1)	
At least one chronic illness, n (%)	16 (41)	24 (42.1)	0.982
Immunosuppression, n (%)	8 (20.5)	7 (12.3)	0.271
Cardiovascular, n (%)	3 (7.7)	11 (19.3)	0.1245
Respiratory, n (%)	5 (12.8)	7 (12.3)	0.811
Renal, n (%)	2 (5.1)	6 (10.5)	0.431
Liver, n (%)	3 (7.7)	1 (1.8)	0.174
At least one organ dysfunction on admission, n (%)	37 (94.9)	49 (86)	0.187
Cardiovascular, n (%)	25 (64.1)	36 (63.2)	0.901
Respiratory, n (%)	34 (87.2)	45 (78.9)	0.284
Neurologic, n (%)	13 (33.3)	15 (26.3)	0.375
Renal, n (%)	9 (23.1)	15 (26.3)	0.752
Delay between ICU admission and fungemia, days, median (IQR)	8 (4–13)	10 (3–21)	0.229
Delay between hospital admission and fungemia, days, median (IQR)	20 (10–29)	21 (11–32)	0.591
Sepsis syndrome, n (%)			0.301
Sepsis	3 (7.7)	11 (19.3)	
Severe sepsis	14 (35.9)	20 (35.1)	
Septic shock	22 (56.4)	26 (45.6)	
SOFA, n (%)			0.004
0–4	3 (7.7)	14 (24.6)	
5–7	6 (15.4)	18 (31.6)	
8–11	12 (30.8)	19 (33.3)	
≥12	18 (46.2)	6 (10.5)	
SOFA score, respiratory, n (%)			0.019
0	4 (10.3)	8 (14)	
1	1 (2.6)	7 (12.3)	
2	8 (20.5)	23 (40.4)	
3	13 (33.3)	16 (28.1)	
4	13 (33.3)	3 (5.3)	
SOFA score, cardiovascular, n (%)			0.015
0	9 (23.1)	19 (33.3)	
1	4 (10.3)	12 (21.1)	
2	1 (2.6)	4 (7)	
3	4 (10.3)	13 (22.8)	
4	21 (53.8)	9 (15.8)	
SOFA score, neurological, n (%)			0.249
0	13 (33.3)	17 (29.8)	
1	5 (12.8)	14 (24.6)	
2	7 (17.9)	8 (14)	
3	4 (10.3)	11 (19.3)	
4	10 (25.6)	7 (12.3)	
SOFA score, renal, n (%)			0.122
0	11 (28.2)	32 (56.1)	
1	8 (20.5)	10 (17.5)	
2	5 (12.8)	5 (8.8)	
3	5 (12.8)	3 (5.3)	
4	10 (25.6)	7 (12.3)	
SOFA score, coagulation, n (%)			0.125
0	19 (48.7)	42 (73.7)	
1	6 (15.4)	3 (5.3)	
2	4 (10.3)	7 (12.3)	
3	6 (15.4)	4 (7)	
4	4 (10.3)	1 (1.8)	
SOFA score, liver, n (%)			0.142
0	26 (66.7)	46 (80.7)	
1	6 (15.4)	6 (10.5)	
2	1 (2.6)	4 (7)	
3	6 (15.4)	1 (1.8)	
Need for mechanical ventilation, n (%)	37 (94.9)	47 (82.5)	0.094
Hypotension, n (%)	25 (64.1)	29 (50.9)	0.208
Presumed source of infection, n (%)			0.573
No clear source	12 (30.8)	20 (35.1)	
Catheter-related	6 (15.4)	15 (26.3)	
Intra-abdominal	8 (20.5)	4 (7)	
Respiratory	6 (15.4)	8 (14)	
Urinary	2 (5.1)	2 (3.5)	
Others	2 (5.1)	4 (7)	
Multiple sources, n (%)	3 (7.7)	4 (7)	
Delay of adequate treatment, n (%)			0.694
<24 h	11 (28.2)	11 (19.3)	
>24 h and ≤48 h	9 (23.1)	14 (24.6)	
>48 h and ≤120 h	12 (30.8)	24 (42.1)	
>120 h or never	7 (17.9)	8 (14)	
Antifungal treatment on the day of blood culture sampling, n (%)	8 (20.5)	8 (14)	0.491
Candida pathogen, n (%)			0.693
Candida albicans	23 (59)	33 (57.9)	
Candida parapsilosis	5 (12.8)	5 (8.8)	
Other fungus	11 (28.2)	19 (33.3)	

*SOFA* Sequential Organ Failure Assessment

On multivariable analysis (Table 5), the only variables independently associated with 28-day mortality were: liver SOFA score ≥3 (odds ratio (OR) 14.35, 95 % CI 1.17–175.6, *p* = 0.037), need for mechanical ventilation (OR 8.86, 95 % CI 1.2–65.24, *p* = 0.032) and ICU admission for medical reasons (OR 3.87, 95 % CI 1.25–11.99, *p* = 0.020). On multivariate analysis SOFA score was the only risk factor for death at 28 days in the subpopulation of patients with ICU-acquired fungemia (*p* = 0,0085).

**Table 5:** Risk factors for death at 28 days for candidemia subpopulation (multivariate analysis)

Variable	Estimate (standard error)	Odds ratio (95 % CI)	*P* value
Delay of adequate treatment			
≤24 h, n (%)	Reference	1	0.8069
>24 h and ≤48 h, n (%)	−0.24 (0.74)	0.79 (0.18; 3.41)	0.7466
>48 h and ≤120 h, n (%)	−0.61 (0.66)	0.54 (0.15; 2.0)]	0.3570
>120 h or never, n (%)	−0.15 (0.90)	0.86 (0.15; 5.14)	0.8717
Medical admission	1.35 (0.57)	3.87 (1.25; 11.99)	0.0197
SOFA score, liver (≥3)	2.66 (1.26)	14.35 (1.17; 175.6)	0.0373
Need for mechanical ventilation	2.18 (1.00)	8.86 (1.2; 65.24)	0.0325

Variables entered into the selection were the Simplified Acute Physiology Score II at ICU admission (per point); medical admission; septic shock at admission; type of ICU (open/closed); Sequential Organ Failure Assessment (*SOFA*) score, coagulation (≥3); SOFA score, liver (≥3); SOFA score, neurological (≥3); SOFA score, renal (≥3); mechanical ventilation and delay of adequate treatment

## Discussion

This prospective, multicenter, observational study discloses that fungemia accounted for 8.3 % of the hospital-acquired BSI in patients admitted to the ICU. The mortality rate in HAF was high (40 %), but delayed adequate antifungal therapy was not an independent risk factor for death. The presence of septic shock and renal dysfunction on ICU admission and renal failure at BSI diagnosis were the only factors associated with fungemia in patients with BSI. In patients with HAF, multivariate logistic regression analysis identified liver SOFA score ≥3, need for mechanical ventilation and ICU admission for medical reasons as independent predictors of 28-day mortality.

Candidemia in critically ill patients is considered to be a severe and life-threatening condition. Recent data show that ICU and hospital mortality rates are usually between 36 % and 61 % [5, 6, 14, 15, 18, 23–26]. In our study, we observed 28 day-mortality of 40.6 % which is in agreement with former findings [27] and is lower than the rates observed in recent Australian [19], Brazilian [26] and Spanish-Italian [18] studies.

Characteristics of bacteremic and fungemic patients at admission are remarkably similar, namely in terms of the comorbidity index, immunosuppression and chronic organ illnesses, and only the presence of septic shock and renal dysfunction on ICU admission and renal failure at diagnosis were more frequent in patients with HA-BSI and fungemia than in those with bacteremia. Although delay in appropriate treatment was significantly longer for HAF, the outcome was similar in patients with fungemia and those with bacteremia.

Kumar et al. [28] observed strong correlation between shorter time to onset of antimicrobial treatment and reduced mortality in patients with septic shock. They showed that over the first 6 hours after the onset of persistent or recurrent hypotension, each hour of delay in initiating effective antimicrobial therapy was associated with a mean reduction in survival of 7.6 %, including the subgroup of patients with fungal infection. Nevertheless, the results of studies addressing the potential benefit of early antifungal therapy in patients with candidemia are conflicting.

In a small prospective multicenter study (n = 46 patients) [14], there was a higher (though not significant, *p* = 0.06) probability of survival among patients receiving early antifungal therapy (within ≤48 hours) compared with those treated 48 hours or more after the diagnosis of candidemia. A few years later, an independent association between delayed antifungal therapy (>48 hours) and in-hospital mortality (hazard ratio (HR) 2.1, 95 % CI 1.0–4.4, *p* = 0.05) was reported by Blot et al. [27]. According to Morrell et al. [29], mortality doubles when antifungal agents are administered ≥12 hours after the collection of the first positive blood culture and in another retrospective analysis of 230 patients with candidemia [30], initiation of fluconazole ≤24 hours after the first positive blood culture was associated with a significantly lower mortality rate. This positive effect of early antifungal therapy on survival was further confirmed in a group of patients with candidemia-associated septic shock who received treatment within 15 hours of blood culture collection [31]. Finally, in 216 patients hospitalized in five teaching hospitals in Italy and Spain (18), who had septic shock attributable to candidemia, adequate antifungal therapy, meaning both infecting organism susceptibility and adequate antifungal dosage within the first 24 hours of culture positivity, was one of the factors associated with 30-day survival.

However, some recent studies did not observe this beneficial association between timing of antifungal therapy and mortality in patients with candidemia [32–34]. Kludze-Forson et al. [35] observed a higher in-hospital mortality rate in patients receiving antifungal therapy within the first 24 hours (50 %) compared to 24–48 hours (28 %) and more than 48 hours (32 %) after blood culture collection. More recently, in a large retrospective study that included 446 patients with candidemia [36], there was neither a significant association between time from positive culture to administration of appropriate antifungal therapy and 30-day mortality, nor between timing of appropriate antifungal therapy and microbiological resolution of *Candida* bloodstream infection. Studies addressing only ICU-acquired candidemia, also failed to show this positive impact [19, 25, 37]. In the study of Marriott et al. [19], mean time to initiation of antifungal therapy was similar for non-survivors and survivors (2.0 ± 1.3 days vs 2.3 ± 1.6 days, *p* = 0.13), and Charles et al. [37] also observed that early therapy (≤48 hours after onset of candidemia) did not improve the outcome of patients with candidemia. Finally, a prospective, Spanish multicenter study showed that inadequate antifungal therapy was a factor independently associated with early mortality (0–7 days) in candidemia, but it had no impact on late mortality (8–30 days) [38].

In our study, four factors may contribute to the absence of impact of delayed adequate antifungal therapy on mortality. First, in critically ill patients, antifungal therapy is more likely to be started earlier than in non-ICU patients and, in fact, 22.9 % of the patients received adequate treatment within the first 24 hours and almost half of them within the first 48 hours after blood culture collection. Second, among critically ill patients any relationship between mortality and initiation of antifungal therapy may be hidden by the power of the patient’s acute illness as a determinant of outcome. Third, only half of our fungemia patients had septic shock at BSI diagnosis and the impact of early therapy is understandably higher in this subgroup of patients. Finally, timing to source control is a possible confounder that could not be analyzed, namely we did not collect data on the presence of a catheter, except on removal if it was deemed the source of the bloodstream infection.

Few prognostic factors have been identified in ICU patients with candidemia. In this cohort, the only independent prognostic factors associated with 28-day mortality were liver SOFA score ≥3, need for mechanical ventilation and ICU admission for medical reasons. Acute severity of illness is one important prognostic factor in candidemia. Among candidemic patients, the severity of organ dysfunction at fungemia onset evaluated by the SOFA score is a risk factor for mortality [39]. In our study, only liver failure was independently associated with 28-day mortality. In *Candida* bloodstream infection, this association between liver disease and outcome was previously reported but only for patients with chronic liver illness, which has a significant HR as an independent risk factor for 30-day mortality (HR 2.15, 95 % CI 1.48–3.13, *p* <0.001) [34]. There is also a relationship between other scores, such as the APACHE II score, and mortality in candidemic patients [14, 23–25, 40]. In our study, we used the SAPS II score but despite being higher in non-survivors (52 vs 47, *p* = 0.095), we could not prove it was an independent risk factor for mortality. A possible explanation is the fact that this score was calculated on admission and may not reflect the severity of the patients at the time of BSI diagnosis.

The need for mechanical ventilation as an independent risk factor for mortality has also been reported by several authors. In a prospective, multicenter, observational French study [5], the use of mechanical ventilation increased the odds of dying in the ICU 2.54 times (95 % CI 1.33–4.82, *p* = 0.0045). A similar result was reported in another large retrospective study (n = 987 patients with candidemia) [41] that showed an independent association between mechanical ventilation and 30-day mortality (OR 2.61, 95 % CI 1.81–3.78, *p* <0.001). An even higher impact was observed in a study with 173 ICU-acquired episodes of candidemia, in which the need of mechanical ventilation was associated with a four times increased risk of death (95 % CI 1.93–8.41, *p* <0.001) [19]. Almirante et al. also reported that intubation (OR 7.5, 95 % CI 2.6–21.1) was associated with higher odds of 30-day mortality in a cohort of 345 patients with candidemia, of whom 33 % were in the ICU at diagnosis [42].

In our cohort, medical patients had a lower probability of survival. Interestingly, the same result was reported by Charles et al. [37] a few years ago in a small study of 51 ICU patients with candidemia, in which medical patients had a higher mortality rate compared to surgical patients (85 % vs 45.2 %) and prior surgery was an independent factor associated with survival (HR 0.25, 95 % CI 0.09–0.67, *p* <0.05). Other authors [19] observed that the chances of dying with candidemia are 6.97 times higher in patients without multi-trauma (95 % CI 1.64–29.67, *p* = 0.009).

Although our study is multicenter, prospective and includes a significant number of patients, it has some limitations. It is not representative of the populations of healthcare systems in the 24 participating countries, and in some countries the number of patients included was very small. Each participating ICU performed investigations and laboratory testing according to their local protocols. The data were entered into the electronic case report form by investigators at each center, which may have increased the risk of inconsistency. Finally, some important variables with potential impact on the outcome were not considered, namely the antifungal agent used, its appropriateness and the timing of central venous catheter removal.

## Conclusion

In summary, this multicenter international study showed that fungi are an important cause of HA-BSI in patients admitted to the ICU. No significant differences were observed between patients with bacteremia and fungemia, except for the presence of septic shock and renal dysfunction on ICU admission and renal failure at diagnosis, which were more frequent in HAF. Fungemia is significantly associated with 28-day all-cause mortality. We were not able to detect an independent association between timing of antifungal therapy and outcome, and only liver failure, need for mechanical ventilation and ICU admission for medical reasons were independent risk factors associated with mortality.

## Key messages

- A fungus is likely to be the cause of a hospital-acquired BSI in patients with septic shock and renal dysfunction on ICU admission or with renal failure at BSI diagnosis
- In patients with hospital-acquired fungemia, the presence of liver failure, the need for mechanical ventilation and ICU admission for medical reasons significantly increase the odds of dying at day 28

## Additional file

Additional file 1: Table S1. Baseline characteristics of patients with fungemia. **Table S2** Characteristics of fungemia episodes at diagnosis. (DOCX 19 kb)

## Abbreviations

APACHE: Acute Physiology and Chronic Health Evaluation
BSI: bloodstream infection(s)
HA-BSI: hospital-acquired bloodstream infection(s)
HAF: hospital-acquired fungemia
HR: hazard ratio
ICU: Intensive Care Unit
IQR: interquartile range
OR: odds ratio
SAPS: Simplified Acute Physiology Score
SOFA: Sepsis-related Organ Failure Assessment
