# Supplementary material for: Characteristics and risk factors for 28-day mortality of hospital acquired fungemias in ICUs: data from the EUROBACT study
Source: Crit Care. 2016 Mar 9;20:53. doi: 10.1186/s13054-016-1229-1 (PMC4784333; doi:10.1186/s13054-016-1229-1)
Supplement: Additional file 1: Table S1. — Baseline characteristics of patients with fungemia. Table S2 Characteristics of fungemia episodes at diagnosis. (DOCX 19 kb) [file 13054_2016_1229_MOESM1_ESM.docx]

**Table S1 - Baseline characteristics of patients with fungemia**

| **Variable** | **ICU acquired fungemia patients n=74** | **Hospital Acquired fungemia patients n=22** | **p=** |
| --- | --- | --- | --- |
| Age, median (IQR) | 61 [44 ; 73] | 62.5 [54 ; 78] | 0.25 |
| Male gender, n (%) | 49 (66.2) | 16 (72.7) | 0.57 |
| SAPS II, median (IQR) | 47 [40 ; 59] | 57 [42 ; 72] | 0.12 |
| Medical admission, n (%) | 47 (63.5) | 14 (63.6) | 0.99 |
| Charlson co-morbidity index, n (%) |  |  | 0.47 |
| 0 | 21 (28.4) | 4 (18.2) |  |
| 1-2 | 29 (39.2) | 8 (36.4) |  |
| ≥ 3 | 24 (32.4) | 10 (45.5) |  |
| At least one chronic illness, n (%) | 23 (31.1) | 17 (77.3) | <.01 |
| Immunosuppression, n (%) | 7 (9.5) | 8 (36.4) | <.01 |
| Cardiovascular, n (%) | 8 (10.8) | 6 (27.3) | 0.05 |
| Respiratory, n (%) | 8 (10.8) | 4 (18.2) | 0.36 |
| Renal, n (%) | 6 (8.1) | 2 (9.1) | 0.88 |
| Liver, n (%) | 3 (4.1) | 1 (4.5) | 0.92 |
| At least one organ dysfunction on admission, n (%) | 68 (91.9) | 18 (81.8) | 0.17 |
| Cardiovascular, n (%) | 45 (60.8) | 16 (72.7) | 0.31 |
| Respiratory, n (%) | 62 (83.8) | 17 (77.3) | 0.48 |
| Neurologic, n (%) | 22 (29.7) | 6 (27.3) | 0.82 |
| Renal, n (%) | 18 (24.3) | 6 (27.3) | 0.78 |
| Septic shock at admission, n (%) | 27 (36.5) | 11 (50) | 0.26 |
| 28-day Mortality, n (%) | 30 (40.5) | 9 (40.9) | 0.98 |

**Table S2 - Characteristics of fungemia episodes at diagnosis**

| **Variable** | **ICU acquired fungemia patients n=74** | **Hospital Acquired fungemia patients n=22** | **p=** |
| --- | --- | --- | --- |
| Delay between ICU admission and BSI, d, median (IQR) | 12 [7 ; 21] | 0 [-1 ; 0] | <.01 |
| Delay between hospital admission and BSI, d, median (IQR) | 18.5 [10 ; 27] | 22.5 [18 ; 38] | 0.18 |
| Delay to positivity of the blood culture sampling, d, median (IQR) | 50.5 [37.7 ; 82.5] | 44.7 [25 ; 73.1] | 0.18 |
| Sepsis syndrome |  |  | 0.60 |
| Sepsis, n (%) | 11 (14.9) | 3 (13.6) |  |
| Severe sepsis, n (%) | 28 (37.8) | 6 (27.3) |  |
| Septic shock, n (%) | 35 (47.3) | 13 (59.1) |  |
| SOFA score |  |  | 0.27 |
| 0-4, n (%) | 14 (18.9) | 3 (13.6) |  |
| 5-7, n (%) | 20 (27) | 4 (18.2) |  |
| 8-11, n (%) | 25 (33.8) | 6 (27.3) |  |
| ≥ 12, n (%) | 15 (20.3) | 9 (40.9) |  |
| SOFA respiratory ≥ 3, n (%) | 38 (51.4) | 7 (31.8) | 0.11 |
| SOFA cardiovascular ≥ 3, n (%) | 33 (44.6) | 14 (63.6) | 0.12 |
| SOFA neurological ≥ 3, n (%) | 24 (32.4) | 8 (36.4) | 0.73 |
| SOFA renal ≥ 3, n (%) 172 (16.2) | 19 (25.7) | 6 (27.3) | 0.88 |
| SOFA coagulation ≥ 3, n (%) | 7 (9.5) | 8 (36.4) | <.01 |
| SOFA liver ≥ 3, n (%) | 5 (6.8) | 2 (9.1) | 0.71 |
| Need for mechanical ventilation, n (%) | 65 (87.8) | 19 (86.4) | 0.85 |
| Hypotension, n (%) | 40 (54.1) | 14 (63.6) | 0.43 |
| Presumed source of infection |  |  | 0.68 |
| No clear source, n (%) | 23 (31.1) | 9 (40.9) |  |
| Catheter-related, n (%) | 18 (24.3) | 3 (13.6) |  |
| Respiratory, n (%) | 11 (14.9) | 3 (13.6) |  |
| Intra-abdominal, n (%) | 8 (10.8) | 4 (18.2) |  |
| Urinary, n (%) | 4 (5.4) | 0 (0) |  |
| Others, n (%) | 4 (5.4) | 2 (9.1) |  |
| Multiple sources, n (%) | 6 (8.1) | 1 (4.5) |  |
| Source control |  |  | 0.54 |
| Not required, n (%) | 41 (55.4) | 13 (59.1) |  |
| Done, n (%) | 29 (39.2) | 9 (40.9) |  |
| Required not done, n (%) | 4 (5.4) | 0 (0) |  |
| Delay of adequate treatment |  |  | 0.35 |
| < 24h, n (%) | 15 (20.3) | 7 (31.8) |  |
| > 24 h and ≤ 48h, n (%) | 16 (21.6) | 7 (31.8) |  |
| > 48h and ≤ 120h, n (%) | 30 (40.5) | 6 (27.3) |  |
| > 120 h or never, n (%) | 13 (17.6) | 2 (9.1) |  |
